# Central tendency measure and wavelet transform combined in the non-invasive analysis of atrial fibrillation recordings

**DOI:** 10.1186/1475-925X-11-46

**Published:** 2012-08-09

**Authors:** Raúl Alcaraz, José Joaquín Rieta

**Affiliations:** 1 Innovation in Bioengineering Research Group, University of Castilla-La Mancha; 2 Biomedical Synergy, Electronic Engineering Department, Universidad Politécnica de Valencia

**Keywords:** Atrial Fibrillation, Central Tendency Measure, Electrical Cardioversion, Electrocardiogram, Wavelet Transform

## Abstract

**Background:**

Atrial fibrillation (AF) is the most common supraventricular arrhythmia in the clinical practice, being the subject of intensive research.

**Methods:**

The present work introduces two different Wavelet Transform (WT) applications to electrocardiogram (ECG) recordings of patients in AF. The first one predicts spontaneous termination of paroxysmal AF (PAF), whereas the second one deals with the prediction of electrical cardioversion (ECV) outcome in persistent AF patients. In both cases, the central tendency measure (CTM) from the first differences scatter plot was applied to the AF wavelet decomposition. In this way, the wavelet coefficients vector CTM associated to the AF frequency scale was used to assess how atrial fibrillatory (*f*) waves variability can be related to AF events.

**Results:**

Structural changes into the *f* waves can be assessed by combining WT and CTM to reflect atrial activity organization variation. This fact can be used to predict organization-related events in AF. To this respect, results in the prediction of PAF termination regarding sensitivity, specificity and accuracy were 100%, 91.67% and 96%, respectively. On the other hand, for ECV outcome prediction, 82.93% sensitivity, 90.91% specificity and 85.71% accuracy were obtained. Hence, CTM has reached the highest diagnostic ability as a single predictor published to date.

**Conclusions:**

Results suggest that CTM can be considered as a promising tool to characterize non-invasive AF signals. In this sense, therapeutic interventions for the treatment of paroxysmal and persistent AF patients could be improved, thus, avoiding useless procedures and minimizing risks.

## Introduction

The Wavelet Transform (WT) has emerged over recent years as one of the most favoured tool by researches for analyzing problematic signals across a wide variety of areas in science, engineering and medicine [1]. It is especially valuable because of its ability to elucidate simultaneously local, spectral and temporal information from a signal in a more flexible way than the short time Fourier Transform (STFT) by employing a window of variable width. This flexible temporal-spectral aspect of the WT allows a local scale-dependent analysis of individual signal features. In this way, both short duration with high frequency details and long duration with low frequency information can be captured simultaneously. Hence, the method is particularly useful for the analysis of transients, aperiodicities and other non-stationary signal features where subtle changes in signal morphology may be highlighted over the scales of interest. Another key advantage of wavelet techniques is the variety of wavelet functions available, thus allowing the most appropriate to be chosen for the signal under investigation. These special features of the WT have been applied to a wide variety of biomedical signals including electromyography, electroencephalography, clinical sounds, respiratory patterns, blood pressure trends, DNA sequences and electrocardiography [2,3]. Moreover, WT has also been successfully employed to solve several problems dealing with electrocardiogram (ECG) signals, such as feature extraction and discrimination between normal and abnormal beats, detection of ventricular late potentials, evaluation of instantaneous changes in heart rate variability, screening of patients with congestive heart failure, etc [4,5]. The present work introduces two different WT applications to ECG signals of patients in atrial fibrillation (AF) that will be next presented.

From an epidemiological point of view, AF is the most common cardiac arrhythmia, affecting almost 5% of the population older than 69 years of age and 8% of the population older than 80 years [6,7]. In this arrhythmia, there exist three main stages in which AF can be placed depending on its usual evolution [8]. Paroxysmal atrial fibrillation (PAF) use to be the first one. In this stage, the arrhythmia terminates spontaneously without the need of medical intervention. The next stage is persistent AF, which requires pharmacological or electrical cardioversion to allow its termination. Finally, the last stage is permanent AF, in which the termination is impossible or is not recommended [6]. This supraventricular arrhythmia is characterized by uncoordinated atrial activation and occurs when the electrical impulses in the atria degenerate from their usual organized pattern into a rapid chaotic pattern [6]. Thus, AF is associated with multiple meandering activation waves propagating randomly throughout the atria [9]. The fractionation of the wavefronts, as they propagate, results in self-perpetuating independent wavelets, called reentries. On the ECG, AF is described by the replacement of consistent P waves by rapid oscillations or fibrillatory (*f *) waves that vary in size, shape, and timing, associated with an irregular ventricular response. Consequently, when AF occurs, a notably disorganized atrial activity (AA) can be observed on the ECG [10].

The associated risks of AF are quite serious because this arrhythmia predisposes to thrombus formation within the atria that can cause stroke or any other thromboembolic events [6]. Since about 18% of PAF patients degenerate into persistent AF in less than 4 years [11], the early prediction of AF maintenance is crucial. Thus, appropriate interventions may terminate the arrhythmia and prevent AF perpetuation. In contrast, the prediction of spontaneous PAF termination could avoid unnecessary therapy, reduce the associated clinical costs and improve the patient’s quality of life. In this sense, one of the WT applications that will be next introduced tries to predict the spontaneous PAF termination.

On the other hand, electrical cardioversion (ECV) is one common therapy in the treatment of persistent AF patients. In contrast to pharmacological cardioversion, ECV is the most effective alternative to revert persistent AF back to normal sinus rhythm (NSR), especially if the arrhythmia has been present for more than 24 hours [12]. Although the ECV success rate is high, AF recurrence is common, especially during the first 2 weeks following the procedure [13]. Moreover, ECV has also the potential of causing severe collateral effects, such as post-shock bradycardia, malignant ventricular arrhythmias, arterial thromboembolism and complications related to anesthesia [12]. Hence, it would be clinically very useful to predict NSR maintenance after ECV, before it is attempted. In this way, the risks of cardioversion could be avoided for those patients with high risk of short-term recurrence and, for the health care provider, clinical costs could be optimized because unproductive treatment time and bed usage could be reduced. As a consequence, other very useful application of the WT should be the prediction of ECV outcome in persistent AF.

For the two aforementioned applications of the WT, i.e., the prediction of spontaneous PAF termination and the prediction of ECV outcome in persistent AF, wavelet analysis was combined with a non-linear approach, using continuous chaotic modelling that summarizes the degree of variability in a signal, such as the central tendency measure (CTM) [14]. More precisely, the CTM from the first differences scatter plot of the wavelet coefficients vector associated to the scale containing the typical AF frequency range, was used to evaluate atrial activity *f * waves organization time course. To this respect, structural changes into surface *f * waves reflect the intraatrial activity organization variation [15]. The analysis of this variation is crucial, because several works have demonstrated a decrease in the number of reentries prior to AF termination. Hence, this decrease will provoke an organization variation in the *f * waves and the atrial activity (AA) will slightly evolve to a more organized pattern before AF termination [16]. Moreover, in the context of ECV, some works have also suggested that NSR maintenance would be more likely in patients who present a highly organized AA, because the more disorganized the AA, the higher the number of propagating wavelets [17] and the larger the atrial volume that could support reentries propagation after the shock [18].

The remaining paper is structured as follows. Section Materials describes the used databases, whereas section Methods explains preprocessing applied to ECG recordings, the proposed algorithm based on WT and CTM to predict AF behavior and the statistical study that was carried out. Section Results summarizes the obtained results, which are next discussed in section Discussion. Finally, section Conclusions presents the concluding remarks that will lead the paper to its end.

## Materials

In this work two databases were employed. First, a set of PAF recordings were analyzed to predict spontaneous termination of AF and, secondly, a set of persistent AF recordings were studied to predict ECV outcome. In the next sub-sections, additional details in this respect can be found.

### Paroxysmal AF database

Fifty Holter recordings of 30 seconds in length and two leads (II and V1) available in Physionet [19] were analyzed. The database included 26 non-terminating PAF episodes (group N), which were observed to continue in AF for, at least, one hour following the end of the excerpt, and 24 PAF episodes terminating immediately after the end of the extracted segment (group T). These signals were digitized at a sampling rate of 128 Hz and 16-bit resolution. Nonetheless, they were upsampled to 1024 Hz in order to allow better alignment for QRST complex subtraction, such as Bollmann et al. suggested [10]. This processing step is necessary to extract the AA from the surface ECG, see section Data preprocessing.

### Persistent AF database

Sixty-three patients (20 men and 43 women, mean age 73.4 ± 9.0 years) with persistent AF lasting more than 30 days, undergoing ECV were followed during four weeks. A standard 12-lead ECG was acquired for each patient during the whole procedure and a segment of 30 seconds in length preceding the cardioversion was extracted from each recording for the analysis. All signals were digitized at a sampling rate of 1024 Hz and 16-bit resolution.

After the ECV, 22 patients (34.93%) maintained NSR during the first month. On the contrary, in 31 patients (49.20%), NSR duration was below 1 month and the remaining 10 (15.78%) relapsed to AF immediately after ECV. These 41 patients constituted the group of AF recurrence. All patients were in drug treatment with amiodarone. The median arrhythmia duration was 10.58 months (range 1–47.22) and echocardiography demonstrated a mean left atrium diameter (LAD) of 45.82 ± 6.93 mm. 20.63% of the patients presented underlying heart disease. No statistically significant differences were found in the aforementioned clinical parameters between the patients who maintained NSR and relapsed to AF.

## Methods

### Data preprocessing

In both databases, lead _*V*1_was chosen for the analysis because previous works have shown that AA is dominant in this lead [20]. This signal was preprocessed using forward/backward highpass filtering with 0.5 Hz cut-off frequency to remove baseline wander, next, lowpass filtering with 70 Hz cut-off frequency was applied to reduce high frequency noise and, finally, notch filtering at 50 Hz was applied to remove powerline interference [21]. On the other hand, reliable analysis of the AA from surface ECG recordings requires that ventricular activity has first been cancelled [10]. Although a variety of different techniques exist for this purpose, an adaptive singular value QRST cancellation template was applied [22].

### Wavelet Transform

From a mathematical perspective, the *wavelet* is a smooth and quickly vanishing oscillating function with good localization in both time and frequency. A *wavelet family*_*Ψa*,*b*_(*t*) is the set of elementary functions generated by dilations and translations of a unique admissible *mother waveletΨ*(*t*) [23], i.e.

(1) $$\Psi_{a,b}(t)=|a{|}^{-\frac{1}{2}}\Psi \left(\frac{t-b}{a}\right),$$

where *ab*∈*ℜa*≠0 are the scale and translation parameters, respectively, and *t* is the time. As *a* increases, the wavelet becomes narrower. Thus, one have a unique analytic pattern and its replications at different scales and with variable time localization.

The Discrete Wavelet Transform (DWT) is the sampled version of the Continuous Wavelet Transform (CWT) in a dyadic grid employing orthonormal wavelet basis functions [23]. Hence, the parameters *a* and *b* are sampled using a logarithmic discretization of the *a* scale (*a*=^2*m*^) and this, in turn, is linked to the steps size taken between the *b* locations. To link *b* to *a*, each location *b*, which is proportional to the *a* scale, is moved in discrete steps (*b*=*n*·^2*m*^). Thus, the discretized mother wavelet is

(2) $$\Psi_{m,n}(k)=2^{-\frac{m}{2}}\Psi (2^{-m}k-n),$$

being *m* and *n* the new scale and translation discrete parameters, respectively, and *k* the discrete time instant. Hence, the wavelet decomposition of the AA signal, _*xAA*_(*k*), can be defined as its correlation with the chosen wavelet family _*Ψm*,*n*_(*k*) for each *m* and *n*, i.e.

(3) $$C_{m}(n)=\underset{k}{\sum }x_{\mathrm{AA}}(k)\cdot \Psi_{m,n}(k).$$

The decomposition results in wavelet coefficients *C*, which depend on scale and position. In fact, a vector of wavelet coefficients _**C***m*_ is obtained for each analyzed discrete scale *m*. The information stored in the wavelet coefficients vectors is not repeated elsewhere and allows the complete regeneration of the original signal without redundancy, because the used discretization of the mother wavelet employs orthonormal basis functions [23].

### Central Tendency Measure

Chaotic equations are sometimes used to generate graphs. Thus, the graph *x*(*n* + 2)−*x*(*n* + 1) versus *x*(*n* + 1)−*x*(*n*) produces a scatter plot of first differences of the data, *x*(*n*) being the value of a time series at time *n*. This plot, centered around the origin, gives a graphical representation of the variability degree in the time series and is useful in modeling biological systems, such as hemodynamics and heart rate variability [14]. With this approach, rather than defining a time series as chaotic or not chaotic, the degree of variability or chaos is evaluated.

The CTM is computed from a first differences scatter plot of the data selecting a circular region of radius *ρ*around the origin, counting the number of points that fall within the radius, and dividing by the total number of points. A low CTM value indicates a large amount of dispersion and a high value indicates concentration near the centre. Given *N* data points from a time series, *N*−2 would be the total number of points in the scatter plot. Then, the CTM can be computed as [14]

(4) $$\mathrm{CTM}=\frac{\overset{N-2}{\underset{i=1}{\sum }}\delta (i)}{N-2},$$

where

(5) $$\delta (i)=\left\{\begin{array}{ll} 1, & \text{if}\;\;\sqrt{{\left(x(i+2)-x(i+1)\right)}^{2}+{\left(x(i+1)-x(i)\right)}^{2}}<\rho ,\\ 0, & \text{otherwise}. \end{array}\right)$$

In the present work, *f * waves organization was estimated by computing the CTM from the first differences scatter plot of the wavelet coefficients vector for the scale containing the typical AF frequency range. For this purpose, a seven-level wavelet decomposition was applied to the AA signal. The seventh discrete scale resulted in a good match to the AA frequency band of interest [24], i.e. 4–8 Hz. In fact, the dominant atrial frequency (DAF), i.e., the highest amplitude frequency, was within this range for all the analyzed patients. Furthermore, previous works have proved that values for this frequency below 4 Hz or above 8Hz are unusual [25].

A good matching between wavelet scale and AF frequency range is relevant to preserve the essential behavior of the arrhythmia. In this respect, the DAF has provided to be a concomitant indicator of atrial refractroriness [26], thus revealing very clinically interesting information about AF progression under different therapies [16]. Remark that for sampling rates different from 1kHz, the seventh wavelet scale could not match properly to the typical AF frequency range and, consequently, the proposed method performance could be notably reduced. Thus, resampling up to 1kHz of new input ECG recordings has to be considered as a previous step before their processing under the proposed method.

### Optimal parameters selection

The proposed method performance depends on the chosen wavelet function and the radius *ρ*selected for computation of CTM. However, there are no established rules for the choice of their optimal values [2]. To this respect, a cautious and still exploratory approach is to test different wavelet families and then to compare their efficiency in the specific problem [27]. Unfortunately, on each electrocardiographic application where the WT has been used, a different wavelet family was chosen [4]. In this study, several orthogonal wavelet families were tested, because only in an orthogonal basis any signal can be uniquely decomposed and the decomposition can be inverted without loosing information [23]. Thus, all the different functions from Haar, Daubechies, Coiflet, Biorthogonal, Reverse Biorthogonal and Symlet wavelet families were tested.

On the other hand, an approach similar to the developed by Hornero *et al*[28] was used to select the optimal *ρ*value. Thus, the CTM was computed for radius values of 0.1, 0.2, …, 10 times the standard deviation of the analyzed data, i.e., the wavelet coefficients vector associated with the seventh discrete scale. Normalizing *ρ* in this way provides translation and scale invariance, in the sense that CTM remains unchanged under uniform process magnification, reduction or constant shift to higher o lower values. To this respect, note that the wavelet vector interesting information is carried by the coefficients temporal variations, rather than its absolute amplitude variations. In fact, the amplitude is only dependent on the similarity between the AA under study and the selected mother wavelet. Hence, amplitude gives no useful information about *f * waves variability.

### Statistical analysis

As the considered number of episodes for both analyzed databases was not notably large, the proposed method was validated by a resubstitution approach, i.e., it was learned from all the available data and then tested on the same set of data. The CTM value, for a specific *ρ* value and wavelet function, providing maximum discrimination between groups, that is, the optimum threshold, was obtained by means of a receiver operating characteristic (ROC) curve. The ROC curve is a graphical representation of the trade-offs between sensitivity and specificity when discrimination threshold is varied. For PAF patients, sensitivity (i.e. the true positive rate) was considered as the proportion of non-terminating PAF episodes correctly discerned, whereas specificity (i.e. the true negative rate) represented the percentage of terminating episodes properly identified. Similarly, for the ECV outcome analysis, sensitivity was the proportion of patients relapsing to AF appropriately classified and specificity was the percentage of patients resulting in NSR after ECV accurately predicted. The total number of PAF patients and ECVs precisely classified was considered as the diagnostic accuracy corresponding to each prediction. The CTM value providing the highest accuracy was selected as optimum threshold. The area under the ROC curve (AROC) was also computed. AROC is a single number which represents a summary of performance. For a perfect test the area is 1, while an AROC of 0.5 represents a worthless test.

To verify representativeness of the used databases and generality of the results obtained by the resubstitution approach, a leave-one-out cross-validation (LOOCV) scheme was also used. In this procedure, the method was trained and tested a number of times equal to the number of patients in the database. In each iteration, all the data, except for a single observation, were used for training and the model was tested on that single observation. In addition, significant differences between terminating and non-terminating PAF episodes and between patients who resulted in NSR and relapsed to AF were evaluated making use of a Student’s *t*-test. All the groups had a normal and homoscedastic distribution as the Shapiro–Wilk and Levene tests proved, respectively. A two-tailed value of statistical significance *p*<0.05 was considered statistically significant.

## Results

### Optimal parameters selection

For each studied wavelet function, CTM values from each patient group in the databases were computed with a *ρ*equal to 3 times the standard deviation of the analyzed wavelet coefficients vector. Preliminary experiments showed successful outcomes with this *ρ* that, later, will be adapted more specifically. Differences between patient groups were evaluated by means of Student’s *t*-test and sensitivity, specificity and accuracy of the classification approach were computed making use of a ROC curve. In all the functions of the same wavelet family, similar statistical significance values and the same sensitivity, specificity and accuracy values were noticed for each analyzed AF scenario. Thus, only the function presenting the lowest *p* value is included in Tables 1 and 2 for each wavelet family. As can also be appreciated in these tables, all the wavelet families reached the same discriminant ability for each analyzed scenario. Moreover, the same patients were incorrectly classified by all the families. Consequently, any wavelet family could be used indistinctly. Nonetheless, both for PAF termination and ECV result predictions, the lowest statistical significance value was noticed for biorthogonal family of order (4,4), such as in previous works [29,30]. Thus, considering that only one wavelet function can be applied to the proposed methodology, the aforementioned wavelet function was selected.

**Table 1:** Mean and standard deviation of CTM values for non-terminating and terminating AF groups, statistical significance (*p* value), sensitivity, specificity, accuracy and result from the LOOCV approach for each studied wavelet family

**Wavelet Family**	**Group N**	**Group T**	***p* value**	**Sensitivity**	**Specificity**	**Accuracy**	**LOOCV**
**(Order)**							
Haar	0.939±0.062	0.795±0.060	<0.001	92.31%	91.67%	92%	92%
Daubechies (5)	0.936±0.060	0.792±0.059	<0.001	92.31%	91.67%	92%	92%
Coiflet (3)	0.931±0.060	0.793±0.046	<0.001	92.31%	91.67%	92%	92%
Biorthogonal (4.4)	0.943±0.054	0.798±0.050	< 0.001	92.31%	91.67%	92%	92%
Reverse Biorthogonal	0.928±0.059	0.789±0.049	< 0.001	92.31%	91.67%	92%	92%
(4.4)							
Symlets (5)	0.931±0.060	0.791±0.058	< 0.001	92.31%	91.67%	92%	92%

**Table 2:** Mean and standard deviation of CTM values for patients relapsing to AF and maintaining NSR during the first month post-cardioversion, statistical significance (*p* value), sensitivity, specificity, accuracy and result from the LOOCV approach for each studied wavelet family

**Wavelet Family (Order)**	**ECVs relapsing to AF**	**ECVs maintaing NSR**	***p* value**	**Sensitivity**	**Specificity**	**Accuracy**	**LOOCV**
Haar	0.698±0.047	0.819±0.071	< 0.001	80.48%	81.81%	80.95%	79.37%
Daubechies (5)	0.693±0.051	0.818±0.069	< 0.001	80.48%	81.81%	80.95%	79.37%
Coiflet (3)	0.688±0.052	0.810±0.066	< 0.001	80.48%	81.81%	80.95%	79.37%
Biorthogonal (4.4)	0.691±0.052	0.812±0.067	< 0.001	80.48%	81.81%	80.95%	79.37%
Reverse Biorthogonal (4.4)	0.690±0.053	0.813±0.067	< 0.001	80.48%	81.81%	80.95%	79.37%
Symlets (5)	0.691±0.052	0.808±0.065	< 0.001	80.48%	81.81%	80.95%	79.37%

After optimal wavelet function selection, the proposed method dependence on the radius *ρ* was investigated following the process described in section Optimal parameters selection. For each tested *ρ*value, statistical differences between terminating and non-terminating PAF episodes and between patients who relapsed to AF and maintained NSR after ECV were evaluated by using Student’s *t*-test. In both cases, statistically significant differences were found for *ρ*values between 2.8 and 5.6. Nonetheless, the lowest *p* value was noticed for *ρ*=3.3 and *ρ*=4 in PAF termination and ECV outcome predictions, respectively. Thereby, the CTM was computed with these *ρ*values in each analyzed AF scenario.

### Classification performance

**Figure 1:**
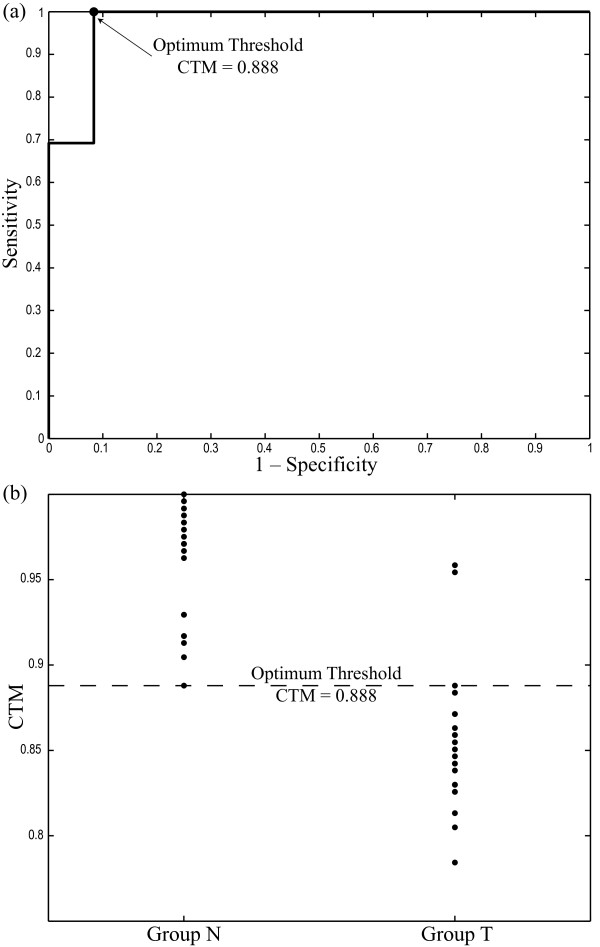
**Results for PAF prediction.(a)** ROC curve constructed with the obtained CTM values for PAF patients from the seventh discrete scale wavelet coefficients of the AA signal. Biorthogonal wavelet family of order (4,4) and a *ρ*value of 3.3 times the standard deviation of analyzed data were used as parameters for CTM computation. The CTM value providing the highest accuracy was selected as optimum threshold, which has been marked with symbol ∙. (b) Classification into terminating and non-terminating PAF episodes.

**Figure 2:**
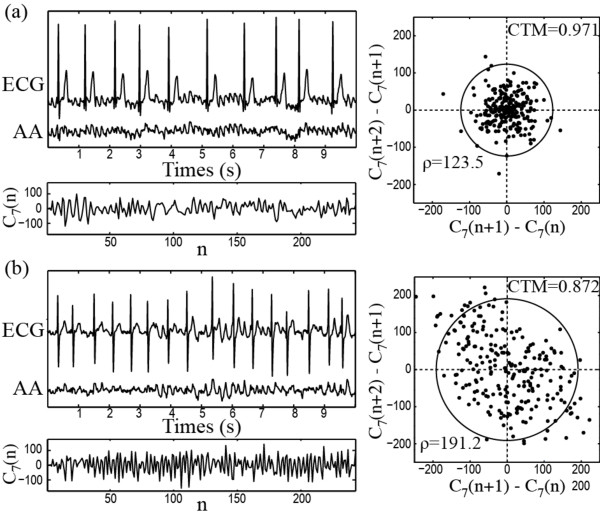
**Representative plots for PAF prediction.** Typical ECG interval together with its extracted AA signal and the wavelet coefficient vector corresponding with the seventh discrete scale together with its scatter plot of first differences for **(a)** a non-terminating and **(b)** other terminating PAF episode.

For PAF termination prediction, CTM values computed with previously described optimal parameters provided sensitivity, specificity and accuracy of 100% (26 out of 26), 91.67% (22 out of 24) and 96% (47 out of 50). In addition, 94% (46 out of 50) of cross-validated grouped cases were correctly identified. The ROC curve provided 0.888 as optimum discrimination threshold between terminating and non-terminating PAF episodes with an AROC of 0.974, see Figure 1(a). Additionally, Figure 1(b) shows that the non-terminating PAF episodes presented higher CTM values (0.963 ± 0.036) than the terminating ones (0.855 ± 0.040). As an example, Figure 2 shows a 10 second-length ECG interval, its extracted AA signal, the wavelet coefficients vector corresponding with the seventh discrete scale and its first differences scatter plot for a typical terminating PAF episode and other non-terminating one. Faster temporal variations within the wavelet coefficients vector and, hence, higher dispersion in scatter plot and lower CTM value can be appreciated for the terminating AF episode.

**Figure 3:**
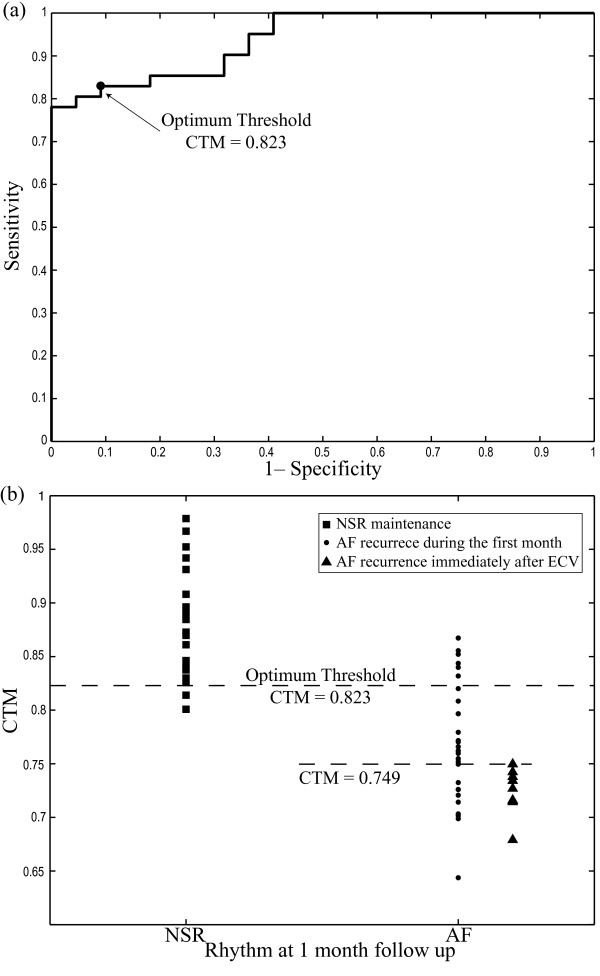
**Results for ECV result prediction.(a)** ROC curve constructed with the obtained CTM values for persistent AF patients from the seventh discrete scale wavelet coefficients of the AA signal. Biorthogonal wavelet family of order (4,4) and a *ρ*value of 4 times the standard deviation of analyzed data were used as parameters for CTM computation. The CTM value providing the highest accuracy was selected as optimum threshold, which has been marked with symbol ∙. **(b)** Classification into patients resulting in NSR and relapsing to AF after 4 weeks following ECV.

**Figure 4:**
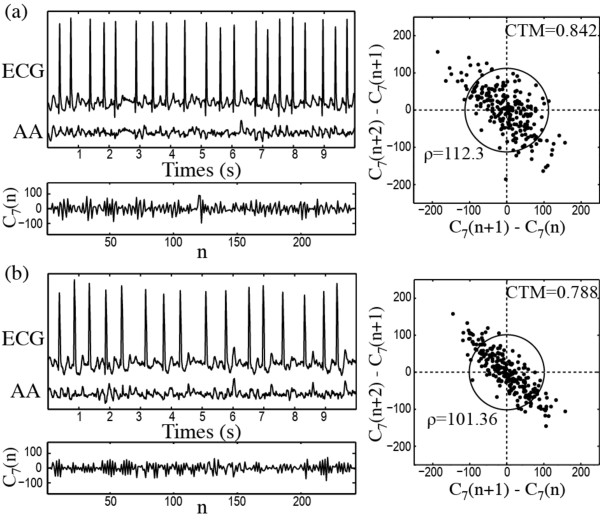
**Representative plots for ECV result prediction.** Typical ECG interval together with its extracted AA signal and the wavelet coefficient vector corresponding with the seventh discrete scale together with its scatter plot of first differences for (**a**) a patient who maintained NSR and (**b**) other relapsing to AF during the first month post-cardioversion

Regarding ECV outcome prediction, the ROC curve provided 0.823 as optimum CTM discrimination threshold, in which 82.93% (34 out of 41) sensitivity and 90.91% (20 out of 22) specificity were obtained, see Figure 3(a). Hence, the ECV outcome in 54 out of 63 patients (85.71%) was correctly predicted, with 82.54% (52 out of 63) of cross-validated grouped cases appropriately identified. An AROC of 0.940 was achieved. Moreover, the patients relapsing to AF presented lower CTM values (0.761 ± 0.054) than those resulting in NSR after one month (0.880 ± 0.050), such as Figure 3(b) shows. As for PAF termination prediction, Figure 4 presents typical ECG intervals, extracted AA signals, wavelet coefficients and scatter plots for a patient who maintained NSR and other relapsing to AF during the first month post-cardioversion of persistent AF. In this case, a higher variation rate within the wavelet coefficients, associated with a lower CTM value, was observed for patients who relapsed to AF.

Other interesting result from Figure 3(b) is that all the patients relapsing to AF immediately after ECV were appropriately identified with a CTM threshold of 0.749. Indeed, these patients presented CTM values (0.727 ± 0.021) lower than the remaining ones who relapsing to AF during the first month post-cardioversion (0.771 ± 0.057), the statistical significance being lower than 0.001. Nonetheless, note that the false positive rate was 32.26% (10 out of 31).

## Discussion

### Comparison with previous works

Non-linear analysis metrics are a valuable tool in the assessment of physiological time series because hidden information related to underlying mechanisms can be obtained [31,32]. To this respect, it has become evident through a number of studies that the cardiac system is non-linear on its behavior [33]. Furthermore, regarding the employment of non-linear metrics, Sample Entropy (SampEn) has been successfully applied to the surface ECG in AF. In this case, SampEn has been computed over the main atrial wave (MAW). The MAW is the fundamental waveform associated to the AA signal and has served as the starting point to estimate non-invasively AF organization. Through the MAW-SampEn strategy, it has been possible to achieve a diagnostic ability of 90% in the prediction of PAF termination [34] and approximately 80% in the prediction of ECV outcome [35]. Similarly, *f * waves regularity has also been assessed through the application of SampEn to the wavelet domain of the AA signal. In this case, the wavelet-SampEn approach reached a discriminant capability of 90 and 82% for PAF termination and ECV outcome predictions, respectively [29,30]. Nonetheless, SampEn might be notably sensitive to the presence of noise [34,36] and spikes [37]. Thus, given that QRST residua are often present in the extracted AA [20,22] and the predictive ability of Wavelet-SampEn could be improved, the analysis of alternative non-linear methods is desirable to develop top performance single classificators of AF organization-related events.

In the present work the CTM from the first differences scatter plot of the wavelet coefficients vector associated to the AF frequency scale has been analyzed. The presented strategy reached the highest diagnostic ability as a single predictor published to date, i.e., 96% and 86% for PAF termination and ECV outcome, respectively. Given that comparison with previous works can be unfair for some cases because different datasets were used, Tables 3 and 4 present the most recently proposed methods, together with relevant information about their validation and performance, to predict PAF termination and ECV outcome, respectively. As aforesaid, AF organization evaluation based on MAW-SampEn or in Wavelet-SampEn provided interesting diagnostic accuracies in both analyzed AF scenarios, but lower than the Wavelet-CTM strategy. In a similar way, the *f * waves amplitude also showed an interesting ability in the prediction of ECV outcome with an accuracy near 80% [38] but, again, lower than this new introduced strategy.

**Table 3:** Comparison between the most recent studies presented to predict PAF termination

**Study**	**Database**	**Short description of methods**	**Diagnostic**
			**accuracy**
This work	Cinc/Challenge 2004 [19]	CTM from the first differences scatter plot of the wavelet coefficient vector associated to the AF frequency scale of the AA	96%
Alcaraz & Rieta 2009 [34]	Cinc/Challenge 2004 [19]	Regularity analysis via SampEn from the MAW of the AA signal	93%
Sun & Wang 2008 [39]	Cinc/Challenge 2004 [19]	Combination of features extracted from the ECG recurrence plot quantification making use of a multilayer perceptron neural network	96%
Alcaraz & Rieta 2008 [29]	Cinc/Challenge 2004 [19]	Regularity analysis via SampEn of time and wavelet domains of the AA	93%
Alcaraz *et al* 2008 [40]	Own Database with 50 episodes: 21 non-terminating and 29 terminating	Analysis of time and frequency parameters obtained from the AA	92%
Nilsson *et al* 2006 [41]	Cinc/Challenge 2004 [19]	Analysis of time and frequency parameters and non-linear indices obtained from the AA	90%
Petrutiu *et al* 2004 [42]	Cinc/Challenge 2004 [19]	Experimental combination of AA peak power evolution within the two last seconds of the episode with the DAF	93%

**Table 4:** Comparison between the most recent studies presented to predict ECV outcome

**Study**	**Database**	**Short description of methods**	**Diagnostic accuracy**
This work	Own database with 63 patients: 31 relapsed to AF, 22 maintained NSR and 10 presented unsuccessful ECV	CTM from the first differences scatter plot of the wavelet coefficient vector associated to the AF frequency scale of the AA	86%
Alcaraz *et al* 2011 [35]	Own database with 63 patients: 31 relapsed to AF, 22 maintained NSR and 10 presented unsuccessful ECV	Combination of *f* waves amplitude and SampEn computed from the MAW of the AA	90%
Alcaraz & Rieta 2009 [38]	Own database with 63 patients: 31 relapsed to AF, 22 maintained NSR and 10 presented unsuccessful ECV	Discriminant model based on time and frequency parameters obtained from the AA	86%
Alcaraz & Rieta 2008 [30]	Own database with 40 patients: 21 relapsed to AF, 14 maintained NSR and 5 presented unsuccessful ECV	Regularity analysis via SampEn of time and wavelet domains of the AA	94%
Watson *et al* 2007 [43]	Own database with 30 patients: 17 relapsed to AF and 13 maintained NSR	Non-parametric combination of several wavelet transform-based statistical markers	93%
Holmqvist *et al* 2006 [44]	Own database with 54 patients: 30 relapsed to AF and 24 maintained NSR	Assessment of the atrial harmonic decay with time-frequency analysis of the ECG	70%
Zohar *et al* 2005 [45]	Own database with 44 patients: 21 relapsed to AF and 23 maintained NSR	Non-deterministisc model based on genetic programming	84%
Berg *et al* 2004 [46]	Own database with 66 patients: 32 relapsed to AF, 22 maintained NSR and 12 presented unsuccessful ECV	Analysis of 3D RR intervals as a quantifier of AF organization	52%

Regarding the frequency domain, a variety of works analyzed the DAF of the AA signal, providing diagnostic abilities in PAF termination prediction between 86% and 90%, depending on the used method for its computation [40,41]. Holmqvist et. al. [44] evaluated a parameter obtained from time-frequency analysis of the AA signals, such as harmonic decay [24], but a low number of patients relapsing to AF after ECV were correctly identified (47%). Finally, in [46], ventricular rhythm was analyzed using three-dimensional RR intervals plots, quantifying clustering of RR intervals, however, only 50% of patients who relapsed to AF during the first 4 weeks following ECV were correctly discerned.

Nevertheless, single parameters combinations and advanced classification tools can be found in the literature to predict AF behavior. Thus, regarding PAF termination prediction, DAF has been combined with other parameters to improve its diagnostic accuracy. To this respect, Petrutiu *et al*[42] studied the AA peak frequency power evolution within the last two seconds before spontaneous PAF termination, reaching thus an accuracy of 93.33%. The same classification result was reported by combining SampEn and WT [29]. In that work, SampEn was used to assess *f * waves regularity from the time and wavelet domains of the AA signal, providing two different and independent classifications, which were combined as a function of the DAF. A slightly higher result (accuracy of 96.67%) was reached by Sun & Wang [39] making use of a multilayer perceptron neural network to combine 11 features extracted from the ECG recurrence plot quantification. With regard to ECV outcome prediction, Watson *et al*[43] examined a variety of wavelet transform-based statistical markers, which obtained a sensitivity of 88% and specificity of 100% by means of a non-parametric classification system. Zohar *et al*[45] developed a non-deterministic model with several parameters as inputs for predicting NSR maintenance after ECV, providing a diagnostic accuracy of 84%. As for PAF termination prediction, the AA signal organization estimation both in time and wavelet domains through SampEn provided an sensitivity of 95% and specificity of 93% [30]. However, it is worth noting that in this study only patients undergoing the first attempt of ECV and, therefore, with AA signals notably organized [47], were analyzed. Finally, recent works have reported that the combination of *f * waves amplitude with the DAF [38] and SampEn [35] computed from the MAW reached a discriminant ability of 86 and 90%, respectively.

Bearing this context in mind, it can be noticed that only complex combinations of single predictors can improve the CTM classification result. Thereby, the proposed metric can be considered as a promising single estimator of PAF termination and ECV outcome prediction, with the additional advantage of a simpler implementation to work in real-time. To this respect, DWT can be computed efficiently with a pyramid filter bank algorithm [23], allowing thus its implementation in real-time [48,49]. In contrast, the classical algorithm proposed in the literature for SampEn computation requires a high execution time, which is not fast enough for online applications [50]. Although faster alternatives for SampEn computation have been recently proposed [50,51], their ability to be implemented in real-time environments has not been proved yet. In addition, accuracy of these new algorithms has not been validated by comparison with the classical SampEn definition. On the other hand, it has to be remarked that the complex combination of multiple parameters or the use of advanced classification techniques, such as in [39] or in [43], makes difficult the clinical interpretation of the results. In this sense, possible clinical meaning of each parameter is blurred within the classification approach.

### Interpretation of results

Taking a specific wavelet decomposition scale, the coefficients vector contains the similarity evolution through time between the analyzed signal and the scaled mother wavelet. A low variability in this time series implies an invariable waveform, regardless of its concrete shape, along the studied time period. In contrast, high variability implies variable waveforms that may, eventually, evolve to a more organized pattern. This fact justifies the results obtained in the prediction of spontaneous PAF termination, where terminating episodes presented wavelet vectors with higher variability than non-terminating ones, see Figure 1. The AA evolution from disorganized *f * waves to organized P waves that takes place in AF recordings prior to its termination may cause this behavior in the wavelet coefficients vector [20].

Regarding ECV result analysis, the presence of more structured *f * waves in organized AA signals [15,20] could justify the obtained results, which show that patients who relapsed to AF presented wavelet coefficients vectors with higher variability than those who remained in NSR, see Figure 3. In fact, patients relapsing to AF immediately after ECV presented, in average, lower CTM values than the remaining ones. These findings, suggesting more organized AA signals in effective cardioversions one month after the procedure, agree with observations obtained from previous works, such as: (i) the higher the AA organization, the higher the success rate in AF cardioversion [17,52], (ii) the higher the AA organization, the lower the energy required for successful cardioversion [18] and (iii) PAF requires less energy for cardioversion than persistent AF [53]. These observations highlight the fact that, when a higher number of reentries are wandering throughout the atrial tissue, a lower probability of successful ECV is obtained. One possible explanation could be that a low degree of AA organization might result in an increased mass of atrial myocardium that is not fully excitable [52].

### Study limitations

The analysis was developed with a limited group of patients. Hence, a larger sample allowing a more rigorous statistical study would be required to provide improved confidence in the robustness of the developed approach. To this respect, wider databases containing non-terminating and spontaneously terminating PAF episodes after different time epochs (ten minutes, half an hour, an hour, ten hours, etc.) and patients who resulted in NSR and relapsed to AF after 3, 6, and 12 months following ECV would be necessary. In addition, the availability of longer PAF recordings would allow to address the interesting question about the time in advance with which spontaneous PAF termination could be predicted. On the other hand, the persistent AF database only included suitable ECV patients following the standard clinical criteria; therefore, it is unknown how the proposed algorithm will behave in patients with adverse clinical predictors, like atrial dilatation, etc., which, by default, are excluded from ECV procedures. Finally, only lead _*V*1_ was analyzed rejecting the possible information contained in the remaining leads. However, for this type of studies, lead _*V*1_seems to be the most suitable lead because significant correlations between the atrial frequency [54] and SampEn [15] obtained from this lead and those obtained from atrial electrograms have been observed.

## Conclusions

The present work has demonstrated that AF organization can be evaluated non-invasively through the application of CTM to the wavelet coefficients vector containing the AF frequency range. This non-linear index has proved to be the most predictive single estimator of spontaneous PAF termination and ECV outcome published to date. Nonetheless, although complex and advanced combinations of other parameters measured from the ECG can improve its diagnostic ability, the proposed algorithm has interesting advantages, including a clear clinical interpretation of the results and the possibility of real-time operation. Hence, its use may lead towards the development of improved therapeutic interventions for the treatment of paroxysmal and persistent AF, since useless procedures could be avoided and the consequent risk for the AF patients could be minimized.

## Competing interests

The authors declare no conflicts of interests.

## Author’s contributions

RA contributed to the development of methods and signal processing tools, evaluated the data, performed analysis and drafted the manuscript. JJR contributed to the design of the study, database collection, signal processing algorithms and writing the manuscript. Both authors read and approved the final manuscript.

## References

[B1] Addison PS The Illustrated Wavelet Transform Handbook. Introductory Theory and Applications in Science, Engineering, Medicine and Finance. Dirac House, Temple Back, Bristol BS1 6BE, 2002 Institute of Physics Publishing, UK

[B2] Rafiee J Rafiee MA Prause N Schoen MP Wavelet basis functions in biomedical signal processing Expert System with Applications 2011 38 5 6190 6201 10.1016/j.eswa.2010.11.050

[B3] Cerutti S, Marchesi C Advanced Methods of Biomedical Signal Processing 2011 John Wiley & Sons. Inc, Hoboken, New Jersey

[B4] Addison PS Wavelet transforms and the ECG: a review Physiol Meas 2005 26 5 R155 R199 10.1088/0967-3334/26/5/R01 16088052

[B5] Acharya R, Suri JS, Krishnan JAESSM Advances in Cardiac Signal Processing 2007 Springer, Berlin Heidelberg, New York

[B6] Fuster V Rydén LE Cannom DS Crijns HJ Curtis AB Ellenbogen KA ACC/AHA/ESC 2006 Guidelines for the Management of Patients with Atrial Fibrillation: a report of the American College of Cardiology/American Heart Association Task Force on Practice Guidelines and the European Society of Cardiology Committee for Practice Guidelines (Writing Committee to Revise the 2001 Guidelines for the Management of Patients With Atrial Fibrillation): developed in collaboration with the European Heart Rhythm Association and the Heart Rhythm Society Circulation 2006 114 7 e257—e354 16908781 10.1161/CIRCULATIONAHA.106.177292

[B7] Miyasaka Y Barnes ME Gersh BJ Cha SS Bailey KR Abhayaratna WP Seward JB Tsang TSM Secular trends in incidence of atrial fibrillation in Olmsted County, Minnesota, 1980 to 2000, and implications on the projections for future prevalence Circulation 2006 114 2 119 25 10.1161/CIRCULATIONAHA.105.595140 16818816

[B8] Gallagher MM Camm J Classification of atrial fibrillation Am J Cardiol 1998 82 8A 18N 28N 10.1016/s0002-9149(98)00736-x 9809897

[B9] Allessie MA Konings K Kirchhof CJ Wijffels M Electrophysiologic mechanisms of perpetuation of atrial fibrillation Am J Cardiol 1996 77 3 10A 23A 10.1016/S0002-9149(97)89114-X 8607387

[B10] Bollmann A Husser D Mainardi L Lombardi F Langley P Murray A Rieta JJ Millet J Olsson SB Stridh M Sörnmo L Analysis of surface electrocardiograms in atrial fibrillation: techniques, research, and clinical applications Europace 2006 8 11 911 926 10.1093/europace/eul113 17043067

[B11] Al-Khatib S Wilkinson W Sanders L McCarthy E Pritchett E Observations on the transition from intremittent to permanent atrial fibrillation Am Heart J 2000 147 142 145 10874276 10.1067/mhj.2000.107547

[B12] Gall NP Murgatroyd FD Electrical cardioversion for AF-the state of the art Pacing Clin Electrophysiol 2007 30 4 554 567 10.1111/j.1540-8159.2007.00709.x 17437583

[B13] Tieleman RG Gelder ICV Crijns HJ Kam PJD Berg MPVD Haaksma J Woude HJVD Allessie MA Early recurrences of atrial fibrillation after electrical cardioversion: a result of fibrillation-induced electrical remodeling of the atria? J Am Coll Cardiol 1998 31 167 173 9426036 10.1016/s0735-1097(97)00455-5

[B14] Cohen ME Hudson DL Deedwania PC Applying continuous chaotic modeling to cardiac signal analysis IEEE Eng Med Biol Mag 1996 15 5 97 102 10.1109/51.537065

[B15] Alcaraz R Hornero F Rieta JJ Assessment of non-invasive time and frequency atrial fibrillation organization markers with unipolar atrial electrograms Physiol Meas 2011 32 99 114 10.1088/0967-3334/32/1/007 21119220

[B16] Bollmann A Lombardi F Electrocardiology of atrial fibrillation. Current knowledge and future challenges IEEE Eng Med Biol Mag 2006 25 6 15 23 17220131 10.1109/emb-m.2006.250504

[B17] Sih HJ Zipes DP Berbari EJ Olgin JE A high-temporal resolution algorithm for quantifying organization during atrial fibrillation IEEE Trans Biomed Eng 1999 46 4 440 450 10.1109/10.752941 10217882

[B18] Calcagnini G Censi F Michelucci A Bartolini P Descriptors of wavefront propagation. Endocardial mapping of atrial fibrillation with basket catheter IEEE Eng Med Biol Mag 2006 25 6 71 78 17220137 10.1109/emb-m.2006.250510

[B19] Goldberger AL Amaral LA Glass L Hausdorff JM Ivanov PC Mark RG Mietus JE Moody GB Peng CK Stanley HE PhysioBank, PhysioToolkit, and PhysioNet: components of a new research resource for complex physiologic signals Circulation 2000 101 23 e215 e220 10.1161/01.CIR.101.23.e215 10851218

[B20] Petrutiu S Ng J Nijm GM Al-Angari H Swiryn S Sahakian AV Atrial fibrillation and waveform characterization. A time domain perspective in the surface ECG IEEE Eng Med Biol Mag 2006 25 6 24 30 17220132 10.1109/emb-m.2006.250505

[B21] Sörnmo L Laguna P Bioelectrical Signal Processing in Cardiac and Neurological Applications 2005 Elsevier Academic Press, 84 Theobald’s Road, London WC1X 8RR, UK

[B22] Alcaraz R Rieta JJ Adaptive singular value cancelation of ventricular activity in single-lead atrial fibrillation electrocardiograms Physiol Meas 2008 29 12 1351 69 10.1088/0967-3334/29/12/001 18946157

[B23] Mallat S A Wavelet Tour of Signal Processing 1999 Academic Press, 84 Theobald’s Road, London WC1X 8RR, UK

[B24] Stridh M Sörnmo L Meurling CJ Olsson SB Sequential characterization of atrial tachyarrhythmias based on ECG time-frequency analysis IEEE Trans Biomed Eng 2004 51 100 114 10.1109/TBME.2003.820331 14723499

[B25] Bollmann A Sonne A Esperer H Toepffer I Langberg J Klein H Non-invasive assessment of fibrillatory activity in patients with paroxysmal and persistent atrial fibrillation using the holter ECG Cardiovasc Res 1999 44 60 66 10.1016/S0008-6363(99)00156-X 10615390

[B26] Capucci A Biffi M Boriani G Ravelli F Nollo G Sabbatani P Orsi C Magnani B Dynamic electrophysiological behavior of human atria during paroxysmal atrial fibrillation Circulation 1995 92 5 1193 202 10.1161/01.CIR.92.5.1193 7648665

[B27] Courdec JP Zareba W Burattini L Detection of abnormal time-frequency components of the QT interval using wavelet transformation technique Proc Comput Cardiol 1997 24 661 664

[B28] Hornero R Abásolo D Jimeno N Sánchez CI Poza J Aboy M Variability, regularity, and complexity of time series generated by schizophrenic patients and control subjects IEEE Trans Biomed Eng 2006 53 2 210 8 10.1109/TBME.2005.862547 16485749

[B29] Alcaraz R Rieta JJ Wavelet bidomain sample entropy analysis to predict spontaneous termination of atrial fibrillation Physiol Meas 2008 29 65 80 10.1088/0967-3334/29/1/005 18175860

[B30] Alcaraz R Rieta JJ A non-invasive method to predict electrical cardioversion outcome of persistent atrial fibrillation Med Biol Eng Comput 2008 46 7 625 35 10.1007/s11517-008-0348-5 18437440

[B31] Pincus SM Goldberger AL Physiological time-series analysis: what does regularity quantify? Am J Physiol 1994 266 4 Pt 2 H1643—56 8184944 10.1152/ajpheart.1994.266.4.H1643

[B32] Richman JS Moorman JR Physiological time-series analysis using approximate entropy and sample entropy Am J Physiol Heart Circ Physiol 2000 278 6 H2039—H2049 10843903 10.1152/ajpheart.2000.278.6.H2039

[B33] Brennan M Palaniswami M Kamen P Do existing measures of Poincaré plot geometry reflect nonlinear features of heart rate variability? IEEE Trans Biomed Eng 2001 48 11 1342 7 10.1109/10.959330 11686633

[B34] Alcaraz R Rieta JJ Sample entropy of the main atrial wave predicts spontaneous termination of paroxysmal atrial fibrillation Med Eng Phys 2009 31 8 917 22 10.1016/j.medengphy.2009.05.002 19501538

[B35] Alcaraz R Hornero F Rieta JJ Noninvasive time and frequency predictors of long-standing atrial fibrillation early recurrence after electrical cardioversion Pacing Clin Electrophysiol 2011 34 10 1241 50 10.1111/j.1540-8159.2011.03125.x 21605132

[B36] Chen W Zhuang J Yu W Wang Z Measuring complexity using FuzzyEn, ApEn, and SampEn Med Eng Phys 2009 31 61 8 10.1016/j.medengphy.2008.04.005 18538625

[B37] Molina-Picó A Cuesta-Frau D Aboy M Crespo C Miró-Martínez P Oltra-Crespo S Comparative study of approximate entropy and sample entropy robustness to spikes Artif Intell Med 2011 53 2 97 106 10.1016/j.artmed.2011.06.007 21835600

[B38] Alcaraz R Rieta JJ Time and frequency recurrence analysis of persistent atrial fibrillation after electrical cardioversion Physiol Meas 2009 30 5 479 89 10.1088/0967-3334/30/5/005 19369714

[B39] Sun R Wang Y Predicting termination of atrial fibrillation based on the structure and quantification of the recurrence plot Med Eng Phys 2008 30 9 1105 11 10.1016/j.medengphy.2008.01.008 18343707

[B40] Alcaraz R Rieta JJ Hornero F Non-invasive characterization of atrial activity immediately prior to termination of paroxysmal atrial fibrillation Rev Esp Cardiol 2008 61 2 154 60 10.1157/13116203 18364184

[B41] Nilsson F Stridh M Bollmann A Sörnmo L Predicting spontaneous termination of atrial fibrillation using the surface ECG. Med Eng Phys 2006 28 8 802 808 10.1016/j.medengphy.2005.11.010 16442328

[B42] Petrutiu S Sahakian AV JNg Swiryn Analysis of the surface electrocardiogram to predict termination of atrial fibrillation: the 2004 computers in cardiology/physionet challenge Proc Comput Cardiol 2004 31 105 108

[B43] Watson JN Addison PS Uchaipichat N Shah AS Grubb NR Wavelet transform analysis predicts outcome of DC cardioversion for atrial fibrillation patients Comput Biol Med 2007 37 4 517 523 10.1016/j.compbiomed.2006.08.003 17011542

[B44] Holmqvist F Stridh M Waktare JEP Roijer A Sörnmo L Platonov PG Meurling CJ Atrial fibrillation signal organization predicts sinus rhythm maintenance in patients undergoing cardioversion of atrial fibrillation Europace 2006 8 8 559 565 10.1093/europace/eul072 16831838

[B45] Zohar P Kovacic M Brezocnik M Podbregar M Prediction of maintenance of sinus rhythm after electrical cardioversion of atrial fibrillation by non-deterministic modelling Europace 2005 7 5 500 507 10.1016/j.eupc.2005.04.007 16087117

[B46] Berg MPVD Noord TV Brouwer J Haaksma J Veldhuisen DJV Crijns HJGM Gelder ICV Clustering of RR intervals predicts effective electrical cardioversion for atrial fibrillation J Cardiovasc Electrophysiol 2004 15 9 1027 1033 10.1046/j.1540-8167.2004.03686.x 15363075

[B47] Alcaraz R Rieta JJ Hornero F Non-invasive atrial fibrillation organization follow-up under successive attempts of electrical cardioversion Med Biol Eng Comput 2009 47 12 1247 55 10.1007/s11517-009-0519-z 19730915

[B48] Zheng H Wu J A real-time QRS detector based on discrete wavelet transform and cubic spline interpolation Telemed J E Health 2008 14 8 809 15 10.1089/tmj.2008.0073 18954252

[B49] Quotb A Bornat Y Renaud S Wavelet transform for real-time detection of action potentials in neural signals Front Neuroeng 2011 4 7 21811455 10.3389/fneng.2011.00007 PMC3139942

[B50] Pan YH Wang YH Liang SF Lee KT Fast computation of sample entropy and approximate entropy in biomedicine Comput Methods Programs Biomed 2011 104 3 382 96 10.1016/j.cmpb.2010.12.003 21208680

[B51] Manis G Fast computation of approximate entropy Comput Methods Programs Biomed 2008 91 48 54 10.1016/j.cmpb.2008.02.008 18423927

[B52] Everett TH Kok LC Vaughn RH Moorman JR Haines DE Frequency domain algorithm for quantifying atrial fibrillation organization to increase defibrillation efficacy IEEE Trans Biomed Eng 2001 48 9 969 978 10.1109/10.942586 11534845

[B53] Lau CP Lok NS A comparison of transvenous atrial defibrillation of acute and chronic atrial fibrillation and the effect of intravenous sotalol on human atrial defibrillation threshold Pacing Clin Electrophysiol 1997 20 10 Pt 1 2442 2452 9358486 10.1111/j.1540-8159.1997.tb06084.x

[B54] Husser D Stridh M Cannom DS Bhandari AK Girsky MJ Kang S Sörnmo L Validation and clinical application of time-frequency analysis of atrial fibrillation electrocardiograms J Cardiovasc Electrophysiol 2007 18 41 6 10.1111/j.1540-8167.2006.00683.x 17229299

